# Posterior predictive checks for the detection of extreme response style

**DOI:** 10.3758/s13428-025-02756-6

**Published:** 2025-07-25

**Authors:** Martijn Schoenmakers, Jesper Tijmstra, Jeroen Vermunt, Maria Bolsinova

**Affiliations:** Department of Methodology and Statistics, Tilburg University, Warandelaan 2, 5037AB Tilburg, The Netherlands

**Keywords:** Bayesian item response theory, Response styles, Extreme responding, Generalized partial credit model, Posterior predictive *p* values, Posterior predictive checks

## Abstract

Extreme response style (ERS), the tendency of participants to select extreme item categories regardless of the item content, has frequently been found to decrease the validity of Likert-type questionnaire results (e.g., Moors, *European Journal of Work and Organizational Psychology*, *21*, 271–298, 2012). For this reason, detecting ERS at both the group and individual levels is of paramount importance. While various approaches to detecting ERS exist, these may conflate ERS with the trait of interest, require additional questionnaires to be administered, or require the use of mixture or multidimensional IRT models. As an alternative approach to detecting ERS, Bayesian posterior predictive checks (PPCs) may be a viable option. Posterior predictive checking offers a highly customizable framework for detecting model misfit, which can be directly applied to frequently used unidimensional IRT models. Critically, the use of PPCs to detect ERS does not require strong assumptions regarding the nature of ERS, such as ERS being a continuous dimension or a categorical trait. In this paper, we thus apply PPCs to a generalized partial credit model to detect model misfit related to ERS on both the group and person levels. We propose various possible PPCs tailored to ERS, which are illustrated in an empirical example, and their performance in detecting ERS is examined under various conditions. Suggestions for practical applications are provided, and avenues for future research are explored.

Likert scales are frequently utilized throughout the social sciences to measure various latent constructs (Nemoto & Beglar, 2014; Van Vaerenbergh & Thomas, 2013). The valid use of Likert scales may be adversely affected by response styles, the tendency of participants to provide a particular response to a question (partially) independent of the question’s content (Falk & Cai, 2016; Van Vaerenbergh & Thomas, 2013). While many response styles exist, extreme response style (ERS) is one of the most frequently studied and encountered response styles. ERS is generally defined as the tendency for participants to prefer responding extremely on item scales, regardless of the item content (Greenleaf, 1992; Van Vaerenbergh & Thomas, 2013). In this paper, we use ERS to denote any deviation by an individual relative to the norm population in how the extreme response categories are used. Note that this could mean either an overuse of the extreme response categories compared to the norm or an underuse of the extreme response categories compared to the norm.

As a response style, ERS can reduce the estimated magnitude of effects, as extreme responding adds construct-irrelevant variance to questionnaire responses (Van Vaerenbergh & Thomas, 2013). Note that the fact that ERS adds construct-irrelevant variance to the responses does not imply that all variance of ERS is irrelevant to the substantive construct. Indeed, correlations between ERS and the trait of interest are frequently found in research (e.g., Batchelor & Miao, 2016; Hamilton, 1968). Nevertheless, ERS adds construct-irrelevant variance to measurement unless the correlation between ERS and the substantive trait is 1 (or –1). As an example of ERS adding construct-irrelevant variance, one study found that failing to account for ERS reduced explained variance from 69.5% to 53.5% (Lau, 2007). In addition to reducing the estimated magnitude of effects such as correlations, ERS may substantially alter conclusions about latent trait differences between groups by biasing estimated group means and variances (Schoenmakers et al., 2023). Consequently, it is important to detect ERS when it is present in data.

Various approaches to detecting ERS in data have been considered in the literature. A straightforward approach is to consider the number of extreme responses as an indicator of extreme responding (Hamilton, 1968). As a downside of this approach, it may become difficult to separate the trait of interest from an extreme responding tendency. For example, a person with an extremely high score on the substantive trait may be flagged as an extreme responder, whereas their high number of extreme responses is caused by their position on the substantive trait rather than any tendency to respond in an extreme manner. To effectively deal with these scenarios, it is crucial to separate the substantive trait value from ERS as cleanly as possible.

One way of separating ERS and the substantive trait is to administer a questionnaire specifically designed to measure ERS in addition to the questionnaire measuring the trait of interest (Greenleaf, 1992). While this option may disentangle ERS and the substantive trait, administering an additional questionnaire to participants may be costly in terms of time and resources. Additionally, detection of ERS after measurement has been completed is impossible using this approach (i.e., ERS has to be considered as a potential source of bias by the researcher before the questionnaire measuring the trait of interest has been administered). As a vast majority of studies do not consider ERS as a potential source of bias before measurement, the applicability of this approach may be limited.

To disentangle substantive traits and response styles without administering an additional questionnaire, many mixture and multidimensional IRT models have been developed. While these models exist, their utilization may require strong assumptions about the nature of ERS, which influence the results obtained. For example, mixture models may assume a categorical ERS trait, where classes of respondents are created based on their extreme response tendency (Alexeev et al., 2011; Rost, 1991). Within classes, every participant is assumed to have exactly the same ERS tendency. Other multidimensional IRT models developed for ERS assume a continuous ERS trait, where every participant potentially has a unique score on ERS (Falk & Cai, 2016; Jin & Wang, 2014; Meiser et al., 2019; Wetzel & Carstensen, 2017). When attempting to test for ERS using these models, it may thus be possible that a researcher finds that an ERS model does not fit the data because the type of ERS in the data does not match the way ERS is modeled, rather than due to a general absence of ERS. Even when only considering multidimensional IRT models, not all models conceptualize ERS in the same way. The differing conceptualizations of ERS can result in different conclusions being drawn and thus warrant additional consideration when implementing a multidimensional IRT model (Schoenmakers et al., 2023). Crucially, the true data-generating model is not known beforehand in practice, and it may not always be realistic to assume that it can accurately be recovered afterwards either (Schoenmakers et al., 2024). The lack of knowledge about the true data-generating model in combination with the difference in outcomes provided by various ERS models creates a pressing need for methods of ERS detection that are not reliant on specific choices regarding the operationalization of ERS.

Ideally, an approach to detecting ERS thus separates ERS from the substantive trait, while not requiring an additional questionnaire to be administered or a specific mixture or multidimensional IRT model to be chosen and fitted. One approach that could be considered for this purpose is the use of Bayesian posterior predictive checks (PPCs). In PPCs, data are replicated using the posterior distribution of model parameters. This replicated data is then compared to observed data on various statistics or discrepancy measures specifically designed to detect misfit related to the phenomenon being investigated. If the model fits the data well, the difference between the replicated data and the observed data on the measure of interest should be slight. If the model does not fit the data well, this should result in larger differences between replicated and observed data (Gelman et al., 1996; Mintz et al., 2020; Rubin, 1984). Comparison of the observed and replicated data can be conducted visually or numerically by computing posterior predictive *p* values (PPP values) for relevant statistics or discrepancy measures.

In general, PPC-based methods offer several advantages. First, PPC-based methods explicitly take the uncertainty regarding item and person parameters into account by utilizing posterior distributions of model parameters to generate replicated data. (Li et al., 2017; Zhu & Stone, 2012). Second, PPC-based methods are flexible, as the researcher can choose different discrepancy measures depending on which aspect of the model they are interested in specifically (Li et al., 2017). For example, researchers interested in misfit related to ERS may compare the observed and expected proportions of extreme responses for a participant. In contrast, researchers interested in acquiescent response style could compare the expected and observed proportions of agreement instead. Due to their flexibility, PPC-based methods can potentially detect misfit related to ERS at both the group and person level and can do so without fitting a mixture or multidimensional IRT model.

When applied to ERS specifically, the advantage of using PPC-based methods is twofold. First, we can now utilize a simple IRT model to test whether there is justification for fitting a more complex ERS model to the data. Second, we do not make a priori assumptions about the structure of the ERS, potentially preventing a scenario where we fit the “wrong” ERS model to the data, find a lack of fit, and then conclude there is no ERS present.

While PPC-based methods have been applied to assess model fit in IRT models in general (e.g., Levy et al., 2009; Sinharay & Johnson, 2003), little research has gone into use of PPC-based methods for detecting response styles specifically. Some notable exceptions are studies where PPCs are utilized to detect heterogeneity in response styles in empirical datasets (Adams et al., 2019; Ames, 2022; Leventhal & Stone, 2018). However, conditions in which PPC-based methods would adequately detect response styles (e.g., sample size requirements, number of items required) were not established in these studies. In addition, PPCs were used to examine the fit of an IRTree model, a mixture partial credit model, and a multidimensional nominal response model in two simulation conditions (Leventhal, 2019). However, this study does not assess the potential of PPC-based methods for detecting ERS directly, and instead assesses the model fit of three potential ERS models. While the above studies thus provide valuable additions to the literature, they do not explore general conditions where certain PPCs are effective in detecting ERS. In addition, PPCs other than the ones proposed in the articles mentioned above may show increased performance in detecting ERS. The current article aims to develop several new tailor-made PPCs designed to detect the presence of ERS, while systematically examining the performance of these specific PPCs in establishing the presence of ERS at both the individual and group levels when utilizing a common unidimensional IRT model under various conditions.

The rest of this article is organized as follows: First, the “Posterior predictive checks” section will describe the statistics and discrepancy measures comparing the observed and replicated datasets. Second, the “Empirical example” section will contain an empirical example of the application of the proposed PPCs. Third, the “Methods” section will discuss the conditions of a simulation study examining the performance of various PPC-based approaches at both the person and group levels. Fourth, the “Results” section describes the results of the simulation study. Finally, the “Discussion” section will discuss the results, limitations of the study, and future recommendations for research and practice.

## Posterior predictive checks

When aiming to detect ERS using PPCs, a researcher typically has a measurement model in mind. While PPCs can readily be adjusted to various measurement models, the generalized partial credit model (Muraki, 1997) is a frequently applied unidimensional IRT model for polytomously scored response data (see Donoghue, 1994; Sahu et al., 2019; Stafford et al., 2019), making it well-suited for the purposes of this study. Equations (1) and (2) present the GPCM for an item with $c$ categories scored from 0 to $c-1$.

1 $$P\left({X}_{in}=x, x>0\right)=\frac{\text{exp}{\sum }_{j=1}^{x}{\alpha }_{i}({\theta }_{n}-{\tau }_{ij})}{1+{\sum }_{k=1}^{c}\text{exp}{\sum }_{j=1}^{k}{\alpha }_{i}({\theta }_{n}-{\tau }_{ij})},$$

where ${X}_{in}$ denotes the observed answer on item $i$ by person $n$, ${\alpha }_{i}$ denotes the slope parameter of item $i$, $\theta$ denotes the latent trait and ${\tau }_{ij}$ denotes the $j$’th (with $j$ ranging from 1 to $c-1$) threshold of item $i$. The probability of obtaining an item score of 0 is given in Eq. (2):

2 $$P\left({X}_{i}=0\right)=\frac{1}{1+{\sum }_{k=1}^{c}\text{exp}{\sum }_{j=1}^{k}{\alpha }_{i}({\theta }_{n}-{\tau }_{ij})}.$$

For the purposes of this paper, four-category items will be considered. Note that the GPCM can easily be generalized to a different number of categories by increasing the number of item thresholds.

A first important decision is the level at which one wants to test for ERS. In this paper, checks for ERS at the person and group levels are both considered. Group-level checks may be interesting for researchers aiming to determine if ERS is present in their data in any form. If ERS is found to be present in the data, a researcher may attempt to correct for the ERS to prevent contamination of the results by using one of the multidimensional ERS models discussed in the previous section or proceed to person-level checks in order to learn more about the ERS that is present. For example, a researcher may be interested in which participants show ERS to gain more insight into the causes or correlates of ERS. Alternatively, if the proportion of participants affected by ERS is small, a researcher may prefer to exclude these participants from the data rather than modeling the ERS statistically, provided this exclusion does not compromise the intended inferences. Since person-level statistics and discrepancy measures may be aggregated to the group level, but group-level statistics and discrepancy measures cannot be disaggregated to the person level, person-level checks will be discussed first.

### Person-level PPCs

When considering PPCs to detect ERS at the person level, examining the number of extreme responses (responses of 1 or 4 in the case of four categories) is a natural approach. However, use of the proportion rather than the number of extreme responses enables a fairer comparison of people in the presence of missing data when considering the variance-based group-level test described later, and has been utilized in previous research. (Leventhal, 2019). Note that on the person-level, use of the number or proportion leads to equivalent results, since the proportion of missing responses of a person is constant across draws. First, we thus count the proportion of extreme responses in the observed data $(E{R}_{obs}^{(n)})$ for every participant $n$, where $n=1,\dots ,N$. After obtaining these proportions of extreme responses, we fit the Bayesian GPCM to the data using Markov chain Monte Carlo (MCMC). This results in $T$ draws from the posterior distribution of the item and person parameters. Using these draws and plugging them into Eq. (1) and (2) to obtain category probabilities, we sample from these category probabilities to generate $T$ replicated datasets. For every person in every replicated dataset, the proportion of extreme responses is calculated $\left(E{R}_{rep}^{(t,n)}\right)$, where

$$t = 1, \dots , T.$$

Generally, a PPP value could be calculated as the proportion of replicated datasets where $E{R}_{rep}^{(t,n)}\ge E{R}_{obs}^{(n)}$. PPP values below .05 (the replicated statistics are nearly always lower than the observed statistic) or above .95 (the replicated statistics are nearly always greater than or equal to the observed statistic) indicate poor model fit (Li et al., 2017). Note that unlike frequentist *p* values, PPP values are not uniformly distributed under the null hypothesis (i.e., there is no ERS present). Instead, PPP values tend to be concentrated around .5 (Meng, 1994; Van Kollenburg et al., 2017). As a consequence, using a .1 cutoff does not imply a type I error rate of .1. Instead, the type I error rate will likely be lower (Wang & Xu, 2021).

A problem exists for the above specification of the PPP value: Since the proportion of extreme responses is a discrete variable with a relatively small number of possible values, the probability of $E{R}_{obs}^{(n)}$ being equal to $E{R}_{rep}^{(t,n)}$ is non-negligible. In cases where $E{R}_{rep}^{(t,n)}=E{R}_{obs}^{(n)}$ , we do not know whether the replicated or observed data are more extreme with regards to the proportion of extreme responses. Hence, we count these cases as .5. Note that assigning a value of .5 to these cases makes the index somewhat more conservative, which is intended behavior in the case where we do not know if $E{R}_{rep}^{\left(t,n\right)}>E{R}_{obs}^{\left(n\right)}$. This results in the PPP value for a person being calculated as

3 $$PP{P}_{ER}^{(n)}={T}^{-1}{\sum }_{t=1}^{T}\left[I\left(E{R}_{rep}^{\left(t,n\right)}>E{R}_{obs}^{\left(n\right)}\right)+\frac{1}{2}I\left(E{R}_{rep}^{\left(t,n\right)}=E{R}_{obs}^{\left(n\right)}\right)\right],$$

where the indicator function $I\left(\cdot \right)$ equals 1 if the condition in brackets is met and 0 otherwise. As the PPP value is two-sided, we take a value above .95 or below .05 to potentially indicate the presence of ERS for a person. Note that a PPP value above .95 indicates fewer observed extreme responses than replicated extreme responses, and a PPP value below .05 indicates more observed extreme responses than replicated extreme responses.

As a second option to detect ERS at the person level, we consider a discrepancy measure to detect ERS. Specifically, the squared difference between the observed and expected number of extreme responses can be compared for the actual and the replicated data. Note that the discrepancy was squared to facilitate aggregation to the group level PPP value discussed later (and that the squaring does not affect the person-level outcomes). We use the number of extreme responses as a proportion of extreme responses to give individuals with less missing data greater impact in the group-level PPP value.

As a first step in calculating the discrepancy measure, we count the number of extreme responses per person in the observed and replicated dataset ($N{E}_{obs}^{(n)}$ and $N{E}_{rep}^{(t,n)}$, respectively). Using the posterior item and person parameter draws, we can calculate the probability of an extreme response for every participant using the GPCM category probabilities described in Eqs. (1) and (2). We calculate the expected number of extreme responses for a person *n* in iteration *t* $E(N{E}^{(t,n)})$ in Eq. (4):

4 $$E\left(N{E}^{(t,n)}\right)={\sum }_{i=1}^{K}\left[P\left({X}_{i}=1|{\theta }_{n}^{(t)},{\tau }_{i}^{\left(t\right)},{\alpha }_{i}^{\left(t\right)}\right)+P\left({X}_{i}=4|{\theta }_{n}^{(t)},{\tau }_{i}^{\left(t\right)},{\alpha }_{i}^{\left(t\right)}\right)\right],$$

where $K$ indicates the total number of items and ${\tau }_{i}^{(t)}$ indicates the vector of item thresholds for item $i$ in iteration $t$. For every draw, we compute ${D}_{obs}^{(t,n)}={\left[N{E}_{obs}^{(n)}-E\left(N{E}^{(t,n)}\right)\right]}^{2}$ and ${D}_{rep}^{(t,n)}={\left[N{E}_{rep}^{(t,n)}-E\left(N{E}^{(t,n)}\right)\right]}^{2}$. Equation (5) presents the PPP value for a person:

5 $${PPP}_{D}^{(n)}={T}^{-1}{\sum }_{t=1}^{T} I\left({D}_{rep}^{(t,n)}\ge {D}_{obs}^{\left(t,n\right)}\right) .$$

Since we are interested in the cases where the replicated discrepancies (the data and expected value both originate from the same GPCM model) are larger than or equally as large as the observed discrepancies (the expected value originates from the GPCM model and the data-generating mechanism is unknown), we use a one-sided PPC with a cutoff of .1 rather than the two-sided test discussed before for $PP{P}_{ER}^{(n)}$. Since the test is now one-sided, cases where ${D}_{rep}^{(t,n)}={D}_{obs}^{\left(t,n\right)}$ result in a PPP value of 1 are not problematic, and the earlier correction for ties between observed and replicated values is thus no longer necessary. As the PPP value is one-sided, we take a value below .1 to potentially indicate ERS for a person. The same considerations and cut-off values hold for the group-level PPP values described below.

### Group-level PPP values

Two group-level PPCs are considered, which aggregate the person-level statistic and the discrepancy measure described above, respectively. For the ${PPP}_{D}^{(n)}$, the aggregation can be achieved by comparing the sum of ${D}_{obs}^{(t,n)}$ over persons to the sum of ${D}_{rep}^{(t,n)}$ over persons for every draw, rather than considering each value separately per person. The group-level PPP value is thus computed in Eq. (6) as:

6 $$PP{P}_{D}={T}^{-1}{\sum }_{t=1}^{T}I\left({\sum }_{n=1}^{N}{D}_{rep}^{\left(t,n\right)}\ge {\sum }_{n=1}^{N}{D}_{obs}^{\left(t,n\right)}\right),$$

where *N* denotes the total number of persons.

To aggregate the $E{R}_{PPP}^{(n)}$ to the group level*,* merely summing over persons for every draw is insufficient, as the overall proportion of extreme responses in a test does not necessarily shift due to the presence of ERS. For example, it may be that there are both participants with a negative ERS tendency who exhibit fewer extreme responses than expected and participants with a positive ERS tendency who exhibit more extreme responses than expected, resulting in an overall unchanged proportion of extreme responses at the group level. To combat this, we instead calculate the variance of the proportion of extreme responses, which the presence of individual differences in ERS will inflate. We thus compare the variance of $E{R}_{obs}^{(n)}$ over persons to the variance of $E{R}_{rep}^{(t,n)}$ over persons, as depicted in Eq. (7).

7 $$PP{P}_{ER}={T}^{-1}{\sum }_{t=1}^{T}I\left[{\sigma }^{2}\left(E{R}_{rep}^{\left(t\right)}\right)\ge {\sigma }^{2}\left(E{R}_{obs}\right)\right].$$

Note that this test for ERS is one-sided, as the PPP value will tend to zero in the presence of ERS.

### Empirical example

To further clarify the method and to obtain realistic item parameters for the simulation study, an empirical example is provided. An example dataset was obtained from the Programme for International Student Assessment (PISA). Specifically, we utilized the reading attitude scale from PISA 2009 (OECD, 2010). The scale consists of eleven four-category items, such as “Reading is one of my favorite hobbies”, and “I read only if I have to” (reverse coded). The response options were “strongly disagree”, “disagree”, “agree”, and “strongly agree”, coded from 1 to 4, where higher scores indicate more positive attitudes towards reading. Questions were answered by participants from 74 countries, with a minimum sample size of 327 and a maximum sample size of 38.058 participants per country.

The primary objective of the empirical example was to demonstrate the proposed method using a real-world dataset. We chose to use the Polish data for this empirical example for several reasons. First, an exploratory analysis detailed later in this example found that ERS was present in the Polish data. Second, the item parameters of the Polish dataset were found to be similar to those of the international sample, as detailed in the second part of the empirical example, making it a somewhat representative example.

An implementation of the Bayesian GPCM based on code by Templin (2022) was fitted to the data using the *cmdstanr* package (Gabry et al., 2024; Stan Development Team, 2024). The code used here and all other code used in the study can be found on OSF at https://osf.io/62bxj/. For the person parameter, we set a prior of $\theta \sim N(\text{0,1})$ as is common in IRT (see e.g., (Luo & Jiao, 2018). Concerning the item parameters, we place a diffuse normal prior on the slope: ${\alpha }_{i} \sim N(1, 5)$. Note that this diffuse prior allows items to have negative slopes. While negative item slopes are typically undesirable, we cannot a priori exclude their existence, as they very well may occur. We thus choose not to truncate the prior to the domain of positive numbers. Rather than modeling item thresholds, we utilize item intercepts for computational convenience (Paganin et al., 2023) and numerical stability in the presence of arbitrarily small slopes. The first item intercept is constrained to zero, and the other three intercepts are modeled using a multivariate normal prior: ${d}_{i}\sim N(\mu ,\Sigma )$. $\mu$ is set to a vector of zeros, and $\Sigma$ is a diagonal matrix with every diagonal entry set to $5$. The model was run using two chains with 1000 burn-in iterations and 2000 sampling iterations per chain.

To assess the convergence of the models, the $\widehat{R}$ statistic proposed by Vehtari et al. (2021) was calculated. Following the author’s recommendations, an $\widehat{R}$ below 1.01 was used to assess the convergence of the chains (Vehtari et al., 2021). In this instance, the model converged with a maximum $\widehat{R}$ of 1.009. The minimum effective bulk sample size was 705.37, and the minimum effective tails sample size was 1552.61. As both effective sample sizes exceeded 400, model inferences were likely to be reliable (Vehtari et al., 2021). First, we computed both the group-level $PP{P}_{D}$ and $PP{P}_{ER}$. Recall that a PPP value below .1 would indicate the presence of ERS at the group level. In the presence of missing data, replicated data required for the PPCs were generated so that missing answers were present in the same locations as they were in the observed data. For the Polish dataset, both $PP{P}_{ER}$ and $PP{P}_{D}$ were estimated to be zero, providing evidence of the presence of ERS at the group level.

To gain further insight into person-level ERS, both $PP{P}_{ER}^{(n)}$ and $PP{P}_{D}^{(n)}$ were computed. If $PP{P}_{ER}^{(n)}$ was below .05 or above .95, this was taken as evidence of ERS (a higher-than-expected number of extreme responses noted as H-ERS for higher ERS and a lower-than-expected number of extreme responses noted as L-ERS for lower ERS) influencing this participant. The same held for the $PP{P}_{D}^{(n)}$ being below .1. Table 1 depicts the agreement between the $PP{P}_{ER}^{(n)}$ statistic and $PP{P}_{D}^{(n)}$ discrepancy measure.

**Table 1:** Agreement between the proposed person-level ERS PPP values

PPP value		$PP{P}_{D}^{(n)}$		
	ERS	L-ERS	Nonflagged	H-ERS
	L-ERS	427	256	0
$PP{P}_{ER}^{(n)}$	Nonflagged	0	3825	53
	H-ERS	0	6	335

*Note.* For the $PP{P}_{ER}^{(n)}$, L-ERS denotes a replicated proportion of extreme responses higher than the observed proportion of extreme responses more than 95% of the time. H-ERS denotes an observed proportion of replicated extreme responses lower than the observed proportion of extreme responses more than 95% of the time. To ascertain the directionality of the ERS flagged by $PP{P}_{D}^{(n)}$ values below .1, we compared the mean expected number of extreme responses over iterations to the observed number of extreme responses. If the mean expected number of extreme responses was higher than the observed number of extreme responses, this indicated L-ERS. If it was lower, this indicated H-ERS

As can be seen in Table 1, $PP{P}_{ER}^{(n)}$ and $PP{P}_{D}^{(n)}$ indices agree on which participants are affected by ERS in the vast majority (94%) of cases. In cases where the PPCs reach differing conclusions, the difference between the PPP values depends on the direction of the ERS. For L-ERS, the difference was frequently caused by the $PP{P}_{ER}^{(n)}$ flagging a participant as being influenced by ERS, while the $PP{P}_{D}^{(n)}$ did not flag the participant. The $PP{P}_{D}^{(n)}$ may thus be a somewhat more conservative PPC compared to the $PP{P}_{ER}^{(n)}$ (256 cases). For H-ERS values, the reverse trend occurred (53 cases).

In practice, a researcher may want to identify characteristics of persons affected by ERS. For example, it may be that gender plays a role for the occurrence of ERS. To study potential relationships between ERS and gender, we first compose a $3*2$ contingency table for both PPP values, with two columns for gender (male = 1, female = 2) and three rows for L-ERS, nonflagged ERS, and H-ERS in Table 2.

**Table 2:** Contingency tables containing percentages for both PPP

	$PP{P}_{ER}^{(n)}$			$PP{P}_{D}^{(n)}$		
	Male	Female	Marginal	Male	Female	Marginal
L-ERS	14%	14%	14%	8%	9%	9%
Nonflagged	82%	76%	79%	87%	80%	83%
H-ERS	4%	10%	7%	5%	11%	8%

To test for a dependence between gender and ERS, omnibus ${\chi }^{2}$ tests for both PPP values were conducted separately. Both ${\chi }^{2}$ tests based on the counts reported in Table 2 revealed significant dependence between gender and ERS tendency at an alpha level of $\frac{0.05}{2}$ $\left(PP{P}_{ER}^{(n)}:{\chi }^{2}\left(2\right)=69.426, p<.001;PP{P}_{D}^{(n)}:{\chi }^{2}\left(2\right)=85.297, p<.001\right)$. In this sample, ERS tendency was thus significantly related to gender, with women generally being more likely to be flagged as ERS responders in general and H-ERS responders in particular compared to men. Note that we merely present this example to illustrate the method, and we make no claims regarding its generalizability.

## Methods

A key aim of the paper at hand was to utilize item parameters that are as realistic as possible when conducting the simulation study. To achieve this aim, we decided to further examine the dataset detailed in the “Empirical example” section. Specifically, we aimed to obtain item parameters for all 74 countries present in the dataset to base our simulation parameters on.

When examining the item parameters across countries, the substantive slope, the ERS slope, and their relative magnitude were of primary interest. These parameters help quantify realistic influences of ERS on the category probabilities. Item intercepts were not of primary interest as we were already interested in comparing conditions where the item intercepts are balanced (i.e., symmetrical item response curves) versus conditions where the item intercepts are unbalanced.

As the presence of ERS is a necessity to obtain realistic simulation item parameters, we first set out to determine in how many of the countries ERS was present in the data. To achieve this aim, we fit the Bayesian GPCM to each country separately. The models were thus estimated separately for each country, with no parameters constrained across countries. Again, an $\widehat{R}$ value below 1.01 was taken to indicate model convergence. A more detailed overview of the process undertaken to achieve convergence is displayed in Appendix 1.

After confirming model convergence, $PP{P}_{D}$ and $PP{P}_{ER}$ were computed in each dataset. Strikingly, both values were zero for all countries, indicating model misfit related to extreme responses in 74 out of 74 countries. One reason for the detection of ERS in every dataset may be the relatively large sample sizes per country (327 to 38,058), which lends an extraordinary amount of power to the ERS tests.

**Fig. 1:**
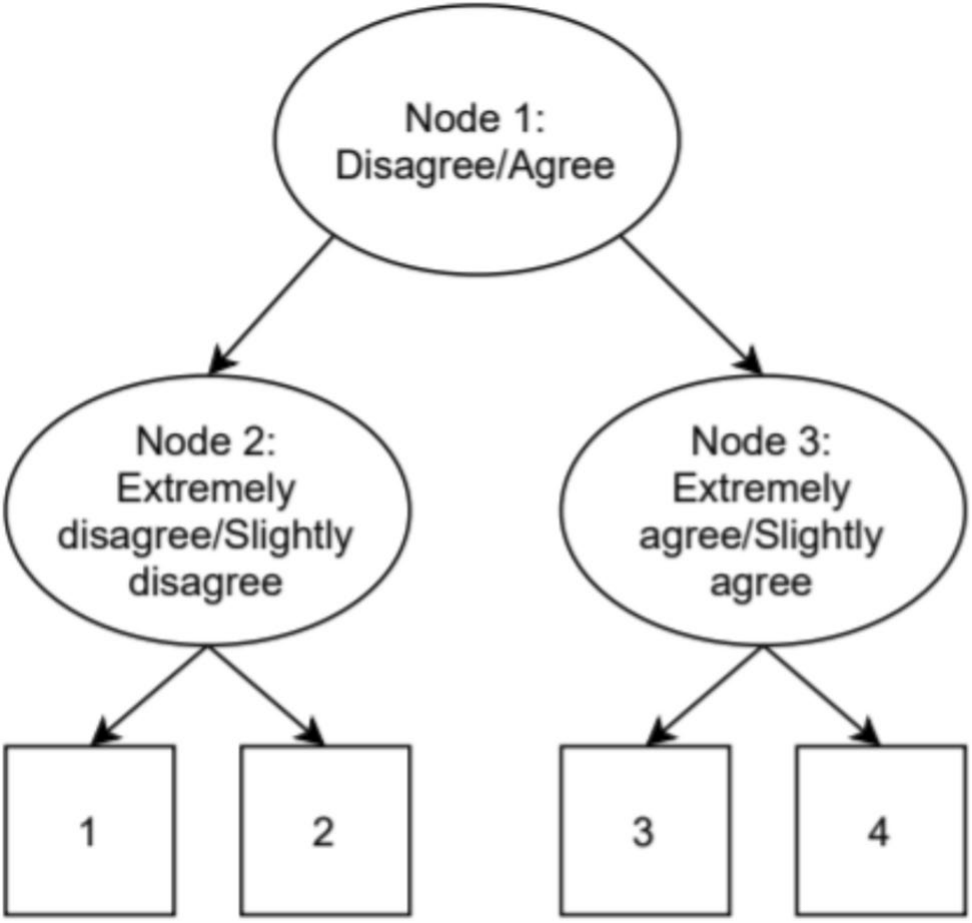
Example of an IRTree decision process for a four-category item

While the use of an ERS model is not necessary to detect ERS, it is necessary when determining realistic item parameters for a simulation and generating data affected by ERS within a simulation. For these parts of the paper, we must thus utilize an ERS model. The first important decision is whether to conceptualize ERS as a continuous or categorical trait. Since assuming a lack of within-class variation in ERS is not theoretically justified (Huang, 2016), this paper will utilize a model that models response styles continuously rather than categorically. In addition to modeling ERS as a continuous trait, the model should be able to accommodate a correlation between ERS and the substantive trait. Finally, the model should offer a conceptualization of ERS where ERS only affects the probability of an extreme response, rather than also affecting the probability of agreeing with an item. Based on these criteria, we chose to use the multidimensional node parameterization of the IRTree model utilized in Schoenmakers et al. (2023). In this model, a four-category item is split into three nodes/pseudo items as depicted in Fig. 1.

The general equation for each node is presented in Eq. (8):

8 $$P({Y}_{im}=1|\theta )=\frac{\text{exp}({\sum }_{v=1}^{2}{\alpha}_{imv}{\theta}_{v} +{d}_{im})}{1+\text{exp}({\sum}_{v=1}^{2}{\alpha}_{imv}{\theta}_{v}+{d}_{im})},$$

where ${\alpha}_{imv}$ denotes the slope parameter of item *i* in node *m* for dimension *v*, ${\theta }_{v}$ denotes the *v*^th^ latent trait, with the first dimension being the substantive trait and the second dimension being the ERS trait, and ${d}_{im}$ denotes the intercept of item *i* in node *m*. Note that the multidimensional IRTree model results in near-identical category characteristic curves to the GPCM if the ERS dimension is removed from the model. This ensures any structural misfit picked up by the PPCs in the simulation study is caused by ERS, rather than being due to fundamental differences between the GPCM and IRTree models in the absence of ERS. Appendix 2 Figs. 3, 4, 5, 6 provide evidence of the practical equivalence of the models by comparing their category characteristic curves over a range of conditions. Note that code for a Bayesian implementation of the models described is also provided at https://osf.io/62bxj/.

The mirt R package (Chalmers, 2012) was used to obtain substantive and ERS slopes for the IRTree model described above. Note that in all cases, both the substantive and the ERS trait were distributed normally with a mean of 0 and a variance of 1. To enable the estimation of a correlation between ERS and the substantive trait, the substantive slope was set equal across node 2 and node 3. In addition, the ERS slope was set to be opposite across node 2 and node 3. Finally, the ERS slope in node 1 was constrained to zero, as ERS should not have an impact on the probability of agreeing with an item. Over all countries, the mean substantive slope in node 1 was found to be 1.92 with a standard deviation of 0.72, the mean substantive slope in node 2 and 3 was found to be 1.14 with a standard deviation of 0.68, and the mean ERS slope in node 2 and 3 was found to be 1.35 with a standard deviation of 0.31. The slope values in the simulation study, detailed later, were based on these mean slope values.

To examine the efficacy of the group- and person-level methods in detecting ERS, a simulation study was conducted. The true-positive rate (TPR; detect ERS when there is ERS in the data) and false-positive rate (FPR; detect ERS when there is no ERS in the data) when detecting ERS were of primary interest here. R version 4.3.3 (R Core Team, 2024) was used to generate all data. First, data without ERS were generated to assess the FPR when no ERS is present. Second, data with ERS were generated to assess the TPR and FPR when ERS is present in the data.

### Null condition

To generate data with no ERS present, the IRTree model described in the “Empirical example” section was utilized to generate data. The ERS score for every participant was set to zero, equivalent to removing the ERS dimension from the model. Based on the empirical example, we chose to use substantive slopes of 1.9 in node 1, substantive slopes of 1.1 in node 2 and 3, and ERS slopes of 1.4 in node 2 and 3 (which will only be relevant for later conditions). Default mean node difficulties of 0 in node 1, – 1 in node 2, and 1 in node 3 were used for every item to create balanced items (items with a 50% probability of agreeing and symmetric category probabilities of [0.2, 0.3, 0.3, 0.2]). To simulate realistic test conditions where not all items are equally difficult, $e$-values equally spaced between – 0.5 and 0.5 were added to all item node difficulties. Within every item, all tree node difficulties thus have the same $e$-value added to them. The sample size was set to 250 for all conditions.

When attempting to detect ERS, several factors may be of interest. First of all, a higher number of items is likely to increase the detectability of ERS at both the group and person level. For this reason, we varied the number of items as 12, 24, and 48.

As a second factor, the number of substantive dimensions was set to 1 or 4. A higher number of substantive dimensions may make it easier to detect ERS, as extreme responding over several dimensions is more easily disentangled from the trait of interest than extreme responding over a single dimension. This is especially the case if all substantive dimensions are uncorrelated. If only a single substantive dimension were present, $\theta \sim N(0, 1)$. When four substantive dimensions were present, ${\varvec{\theta}}\sim N(0,\Sigma )$, with $\Sigma$ being set to a 4x4 identity matrix. Note that we chose to generate the multiple substantive traits as uncorrelated to each other to maximize the difference to the condition where there is only a single substantive trait (which can alternatively be conceptualized as multiple traits with a correlation of 1 to each other). In general, we would thus expect conditions with multiple traits to become more similar to the condition with a single substantive trait as the correlation between the traits approaches 1 (or – 1). As a situation with four substantive dimensions and 12 items is unlikely to occur in practice, we did not consider this condition. For the other two test lengths, items were split equally over all substantive dimensions present.

As a final factor, we shifted all mean item difficulties by a value of zero or – 1. Shifted item difficulties may lead to ERS being more difficult to detect (see e.g., Merhof et al., 2024) for two reasons. First of all, shifted item difficulties will result in items less matched to the mean ability of participants, reducing the information value of each item. Second, a shift in item difficulties results in more extreme answers being given due to the substantive trait, which may obfuscate the ERS dimension when present.

All in all, this leads to 3 (number of items) * 2 (difficulty shift) = 6 conditions when there is a single substantive dimension in the data, and 2 (number of items) * 2 (difficulty shift) = 4 conditions when there are four substantive dimensions in the data, for a total of 10 null conditions.

To estimate the GPCM, we again utilized the cmdstanr package (Gabry et al., 2024). The number of chains, burn-in iterations, sampling iterations, convergence assessment, and priors used in the case of a single substantive dimension were identical to the empirical example. In the case of multiple substantive dimensions, a $LKJ(1)$ prior (Joe, 2006; Lewandowski et al., 2009) was used for the covariance matrix of the substantive traits. The $LKJ(1)$ prior is a uniform prior over the space of all positive definite matrices with unit diagonal. All other setting were the same as the empirical example. As outcomes, the FPR in detecting ERS was calculated at the person and group levels. All conditions were replicated 200 times to achieve reasonable precision around the reported outcomes in this paper.

### Experimental condition

When generating data containing ERS, we once again utilize the IRTree model described in the “Models” section with the same item parameters as described before. Normally, ERS would be sampled from a multivariate standard normal distribution as an additional continuous latent trait. A downside of this approach would be that the TPR and FPR of the method to detect ERS at the person level become difficult to establish. After all, a participant with a ERS value of 0.01 is technically affected by ERS, but it would hardly be reasonable to expect any ERS detection method to pick up on this. To remedy this issue, we generated ERS as discrete values ranging from – 3 to 3 in steps of 0.5 instead of modelling ERS as an additional continuous latent trait, allowing the TPR to be studied for different levels of ERS. The probability of a participant being assigned a certain ERS value was proportional to the normalized density of a standard normal distribution at each ERS value. Note that the range of – 3 to 3 for the ERS values was chosen as preliminary investigation showed this range did not overly shrink the item slopes of the ERS dimension when fitting the ERS IRTree model, as opposed to narrower ERS ranges. When generating ERS this way, we retain a straightforward interpretation of TPR and enable an evaluation of FPR at the person level in the experimental condition.

As conditions, all factors described in the null condition were retained, again resulting in ten conditions. As outcomes, the TPR and FPR of the methods were of interest. At the group level, TPR was calculated as the proportion of replications where the group-level PPP value exceeded the critical value. A FPR could not be established, as all datasets were affected by ERS. At the person level, the TPR was calculated at each nonzero ERS value as the proportion of people flagged by the PPC. The FPR was calculated as the proportion of flagged people (aggregated over all replications) with an ERS value of zero.

## Results

### Null condition results

Table 3 presents the FPRs when attempting to detect ERS in a condition where no ERS is present at both the group and person level. Concerning the group level, the FPR for detecting ERS remains at or below .05 in all but one condition. A remarkable exception to the near-zero FPR is the higher FPR when using the $PP{P}_{ER}$ in conditions where a difficulty shift is present, while multiple substantive dimensions are present. Notably, this effect does not occur for the $PP{P}_{D}$. A complete overview of the group-level PPP values in the null condition is available in Appendix 3, Figs. 7 and 8.

**Table 3:** FPR for detecting ERS in the null conditions

Factors			Group-level PPP values		Person-level PPP values	
$V$	${\beta }_{+}$	$K$	$PP{P}_{ER}$	$PP{P}_{D}$	$PP{P}_{ER}^{(n)}$	$PP{P}_{D}^{(n)}$
1	0	12	.00	.00	.03	.02
		24	.00	.02	.05	.02
		48	.00	.02	.04	.03
	1	12	.04	.01	.03	.01
		24	.01	.02	.04	.02
		48	.00	.01	.02	.02
4	0	24	.03	.01	.04	.02
		48	.00	.02	.02	.02
	1	24	**.11**	.02	.02	.01
		48	.03	.01	.01	.01

*Note.* $V$ denotes the number of substantive dimensions, ${\beta}_{+}$ denotes the constant added to all item node difficulties and $K$ denotes the number of items. Values above .05 are marked in bold

When considering results at the person level, it can be seen that the performance of the methods concerning FPR is satisfactory, with all FPR values remaining at or below .05 in all conditions. Contrary to the group-level results, the introduction of a difficulty shift does not seem to adversely influence the FPR when using the $PP{P}_{ER}^{(n)}$. As all FPR are well within statistical error margins of each other, we do not interpret the results of factors here. Nevertheless, use of the $PP{P}_{D}^{(n)}$ appears to produce a FPR that is equal or lower than the FPR of the $PP{P}_{ER}^{(n)}$ in all conditions. A complete overview of the person-level PPP values in both the null and experimental conditions can be found in Appendix 4, Figs. 9, 10, 11, 12.

### Experimental condition results

Due to the way ERS was generated in this study, it was possible to obtain person-level FPRs in the experimental condition, which are displayed in Table 4. Overall, the FPR of the approach remains below .05 in all but one condition. When the number of substantive dimensions is 1, there is no threshold shift, and the number of items is 24, the $PP{P}_{ER}^{(n)}$ shows a very mildly inflated FPR rising to .06. Generally, adding more substantive dimensions seems to somewhat decrease the FPR when using the $PP{P}_{ER}^{(n)}$. The trend of using $PP{P}_{D}^{(n)}$ resulting in a somewhat lower FPR than using the $PP{P}_{ER}^{(n)}$ continues.

**Table 4:** FPR for detecting ERS at the person level in the experimental conditions

$V$	${\beta}_{+}$	$K$	$PP{P}_{ER}^{(n)}$	$PP{P}_{D}^{(n)}$
1	0	12	.04	.02
		24	**.06**	.03
		48	.05	.04
	1	12	.04	.02
		24	.04	.02
		48	.03	.03
4	0	24	.04	.02
		48	.04	.03
	1	24	.02	.02
		48	.02	.01

*Note.* $V$ denotes the number of substantive dimensions, ${\beta }_{+}$ denotes the constant added to all item node difficulties, $K$ denotes the number of items, and FPR is the false-positive rate. Values above .05 are marked in bold

As the final outcomes, we present the TPR in detecting ERS at the group and person levels. At the group level, it was found that the TPR was equal to one across all conditions when using either PPC. We thus do not display a table or discuss these results further here.

**Fig. 2:**
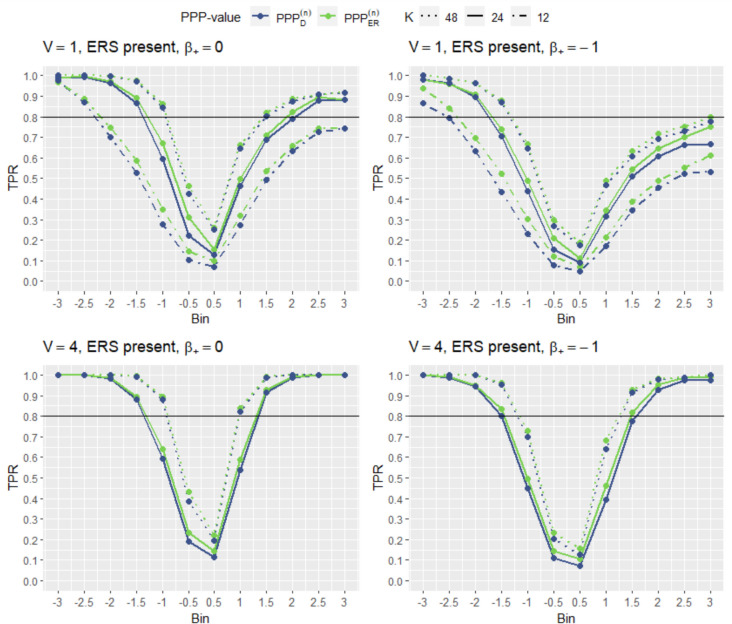
TPR of the person-level PPCs in the experimental condition. *Note.* $K$ is the number of items, $V$ denotes the number of dimensions, and ${\beta }_{+}$ denotes the constant added to all item node difficulties

**Fig. 3:**
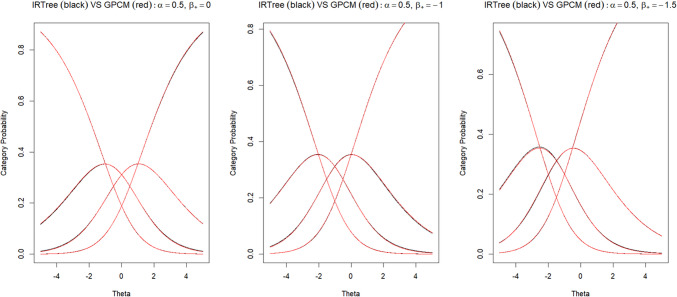
Category probabilities of the IRTree and GPCM models when slopes of all items are 0.5. Note. $\alpha$* indicates the slope for all items, *${\beta}_{+}$* indicates the constant added to all item difficulties*

**Fig. 4:**
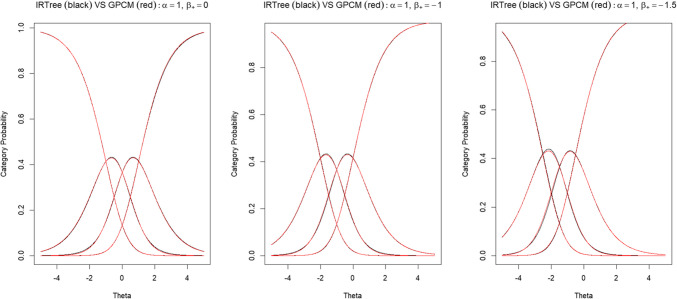
Category probabilities of the IRTree and GPCM models when slopes of all items are 1*. Note.* $\alpha$ indicates the slope for all items, ${\beta}_{+}$ indicates the constant added to all item difficulties

**Fig. 5:**
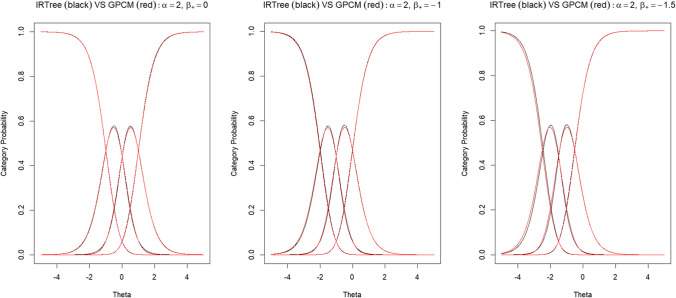
Category probabilities of the IRTree and GPCM models when slopes of all items are 2. Note. ${\alpha}$ indicates the slope for all items, ${\beta}_{+}$ indicates the constant added to all item difficulties

**Fig. 6:**
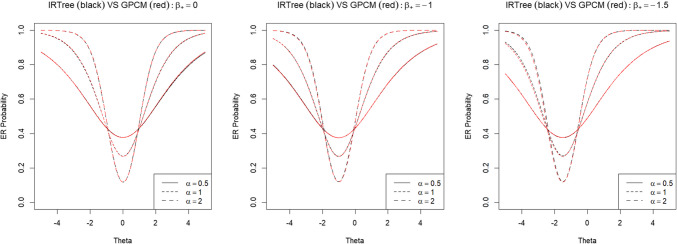
Extreme response probabilities of the IRTree and GPCM models. *Note.* $\alpha$ indicates the slope for all items, ${\beta}_{+}$ indicates the constant added to all item difficulties

**Fig. 7:**
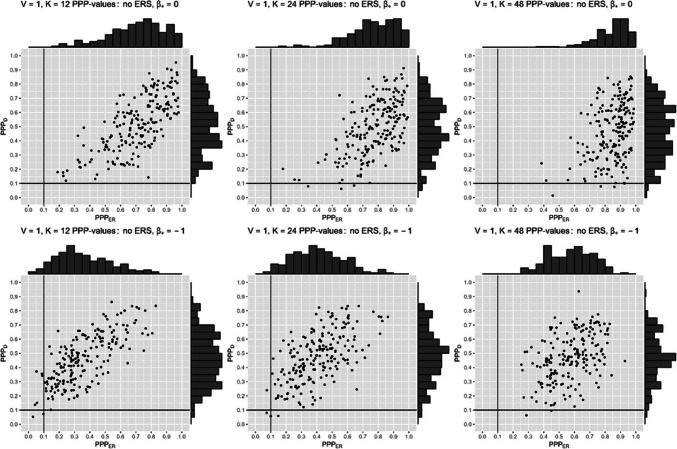
Distribution of the group-level PPP values in the null condition with one substantive dimension present. *Note.* $V$ denotes the number of substantive traits, $K$ is the number of items, and ${\beta}_{+}$ is the constant added to the difficulty parameters

**Fig. 8:**
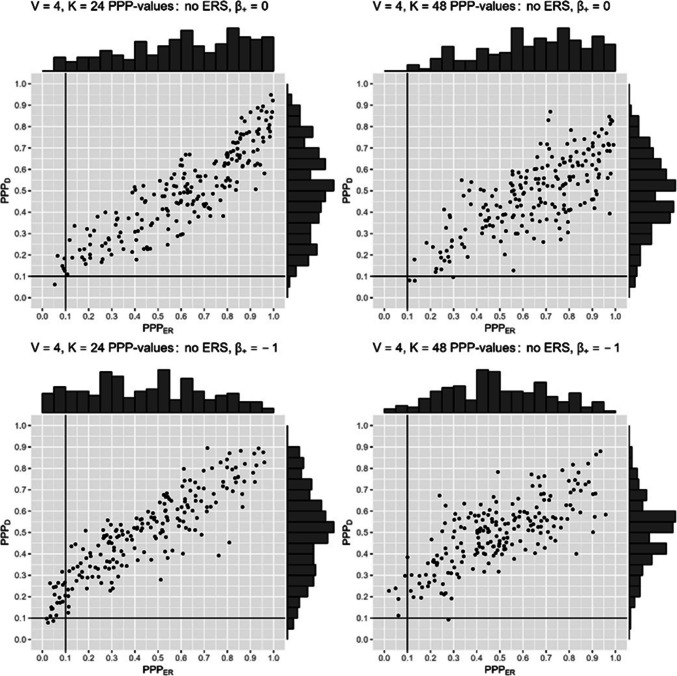
Distribution of the group-level PPP values in the null condition with four substantive dimensions present*. Note.* $V$ denotes the number of substantive traits, $K$ is the number of items, and ${\beta}_{+}$ is the constant added to the difficulty parameters.

**Fig. 9:**
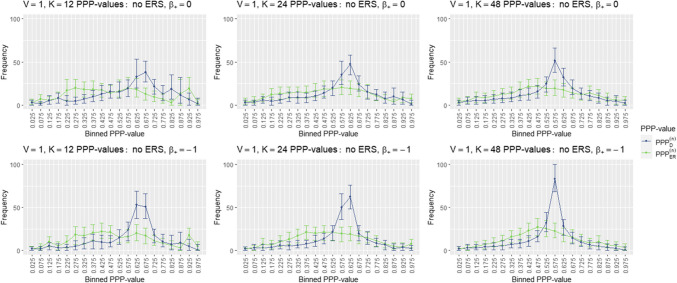
Distribution of the person-level PPP values in the null conditions with a single substantive dimension present. *Note.* Error bars indicate the 95% highest density interval over 200 replications*.* $V$ denotes the number of substantive traits, $K$ is the number of items, and ${\beta }_{+}$ is the constant added to the difficulty parameters.

**Fig. 10:**
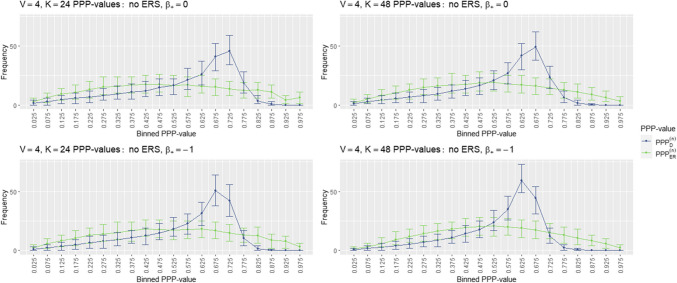
Distribution of the person-level PPP values in the null conditions with four substantive dimensions present. *Note.* Error bars indicate the 95% highest density interval over 200 replications*.* $V$ denotes the number of substantive traits, $K$ is the number of items, and ${\beta }_{+}$ is the constant added to the difficulty parameters.

**Fig. 11:**
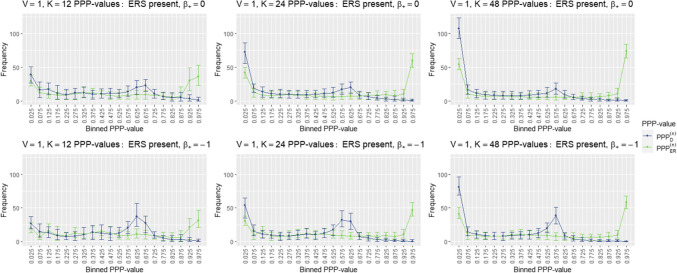
Distribution of the person-level PPP values in the experimental condition with one substantive dimension present. *Note.* Error bars indicate the 95% highest density interval over 200 replications*.* $V$ denotes the number of substantive traits, $K$ is the number of items, and ${\beta }_{+}$ is the constant added to the difficulty parameters.

**Fig. 12:**
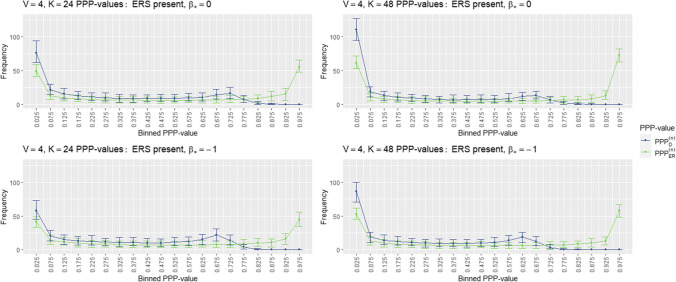
Distribution of the person-level PPP values in the experimental condition with four substantive dimensions present. *Note.* Error bars indicate the 95% highest density interval over 200 replications*.* $V$ denotes the number of substantive traits, $K$ is the number of items, and ${\beta }_{+}$ is the constant added to the difficulty parameters

The TPR (the probability of a person with a non-zero ERS value being flagged as an ERS responder) of detecting ERS at the person level is displayed in Fig. 2. In the top two graphs in Fig. 2, two effects of factors on TPR become apparent for conditions with a single substantive dimension. First of all, increasing the number of items greatly enhances the TPR of the method. Naturally, increasing the number of items gives more information about the participant, which enhances the ability to detect person-level misfit. Second, introducing a difficulty shift decreases TPR of the method for all considered values of ERS. This is likely due to the fact that administering items too easy or too difficult for a population decreases information regarding the persons. In addition, the increased number of extreme responses naturally occurring due to the substantive latent trait may obscure ERS respondents.

Another noticeable trend is an asymmetry in the ease of detecting ERS, with negative ERS values appearing easier to detect than positive ERS values. The fact that positive ERS is more difficult to detect than negative ERS may be due to a decrease in response pattern variability when positive ERS is present (i.e., participants are more likely to answer nearly all 1’s or nearly all 4’s). When a response pattern with low variability occurs, ERS detection methods may have difficulty distinguishing between the response pattern being caused by extreme ERS trait values versus extreme substantive trait values. This also explains why the detectability of positive ERS values does not go to 1, even for very extreme ERS values (3) and a very long unidimensional questionnaire (48 items), Finally, it explains why the asymmetry between the detectability of positive and negative ERS values seems to worsen somewhat when a difficulty shift is introduced. After all, there will be even more naturally occurring extreme response patterns in this scenario. A post hoc exploratory analysis indeed revealed that when considering very extreme positive ERS values and a difficulty shift, a notable proportion of respondents obtained a response pattern where (nearly) all answers are extreme, lending further support to this explanation. Finally, using $PP{P}_{ER}^{(n)}$ results in slightly more power to detect ERS at the person level than using the $PP{P}_{D}^{(n)}$, especially in conditions where the number of items is below 48.

To illustrate the effect of incorporating additional substantive dimensions into the data, the bottom two graphs in Fig. 2 display the TPR when using person-level PPCs with four substantive dimensions present. In general, the TPR of the ERS detection methods is enhanced by the presence of multiple dimensions, even when the number of total items remains the same. Additionally, the asymmetry in detecting positive versus negative ERS is significantly reduced. The fact that positive ERS values are more detectable when multiple substantive dimensions are present may be due to the decreased likelihood of extreme response patterns (nearly all 1’s or all 4’s) being solely caused by the substantive traits without any influence of positive ERS values. Again, using the $PP{P}_{ER}^{(n)}$ results in slightly more overall power to detect ERS at the person level than using the $PP{P}_{D}^{(n)}$, although the difference is minor.

## Discussion

The present study aimed to investigate the efficacy of PPC-based approaches in detecting ERS under various conditions. To achieve this aim, the TPR and FPR of two PPC-based ERS detection methods were evaluated at both the group and individual levels.

At the group level, the FPR of both approaches remained below the .05 mark in all but one condition. Notably, the $PP{P}_{ER}$ approach resulted in a FPR of .11 when 4 substantive dimensions and a difficulty shift were present. As the $PP{P}_{D}$ did not seem to struggle in these conditions (and the TPR was 1 in all conditions for both PPP values), we generally recommend that researchers use the $PP{P}_{D}$ to detect ERS at the group level in practice.

While a FPR below .05 may seem desirable, a FPR of .05 is frequently desired to prevent the method from being overly conservative. Conservativeness of PPCs has been noted in previous literature, and several remedies, such as posterior calibration, are available (Van Kollenburg et al., 2017). These methods work by replicating another large number (e.g., 1000) datasets per replicated dataset to obtain a distribution of the PPP values, which is then used to pick a cut-off value that approximately results in the desired type I error rate. As calibration leads to a vast increase in computation time and the TPR at the group level did not seem to be adversely affected by the low FPR rates (even reaching 1 in all conditions), we did not utilize these methods in this paper. Researchers nevertheless wishing to increase TPR at the group level may consider utilizing some type of calibration. As an alternative to calibration, a researcher may also consider using cutoffs different from the .05/.95 used in this paper. Based on our results, stricter cutoffs (e.g., .025/.975) would not be recommended, as the FPR already was quite low and a stricter cutoff would further decrease the TPR. A more liberal cutoff (e.g., 0.1/0.9) may be considered, but this could very well increase the FPR above acceptable values in at least some conditions. As a final note, the presence of asymmetric item intercepts (i.e., item intercepts not resulting in symmetrical item response curves) may inflate the FPR when using the $PP{P}_{ER}$, but not when using the $PP{P}_{D}$. We thus recommend researchers that are likely to encounter such asymmetric intercepts (e.g., researchers utilizing depression questionnaires) to utilize the $PP{P}_{D}$.

At the individual level, the FPR of both approaches remained at or below 0.05. While the conservativeness of PPCs did not reduce the TPR at the group level, the same does not hold for the individual-level TPR. Researchers may thus consider calibration of the PPCs or using a more conservative threshold to establish the presence of ERS.

Several factors influenced the ease of detecting ERS at the person level. Higher magnitudes of ERS, a higher number of test items, and an increased number of substantive dimensions all led to an increase in TPR. The finding that increasing the number of substantive dimensions may ease the detectability of response styles is in line with previous literature (e.g., Bolt & Newton, 2011). A notable finding in this study was the asymmetry in ease of ERS detection when only a single substantive dimension is present: positive ERS values seem more difficult to detect than negative ERS values of the same magnitude in this scenario. This asymmetry is diminished when multiple substantive traits are measured simultaneously. While the present study illustrated this effect when all substantive traits are uncorrelated, it is important to note that (strongly) correlated substantive traits may lead to a less profound increase in ERS detectability than the one presented in this paper. Researchers aiming to measure strongly correlated traits may thus consider adding more items to their measurement, while being aware that even that might not fully remedy the detection issue.

When aiming to detect ERS at the person level using PPCs in conditions similar to those examined in this paper, we make several recommendations. To better interpret the values of ERS in these recommendations, recall that $ERS\sim N(\text{0,1})$ in this study. In the case of a single substantive dimension and 12 items, only negative ERS values of – 2.5 or lower can be reliably (i.e., a TPR of .8) detected. For 24 items, ERS values at or below –1.5 or 2 and above can reliably be discerned. For 48 items, ERS values at or below – 1 or 1.5 and above were detectable. Note that the number of necessary items may increase further still if the item difficulty is not well-matched to the ability of the population.

As most questionnaires in psychology are not nearly 48 items long, measuring multiple substantive latent traits simultaneously with shorter questionnaires per trait (e.g., the Big Five personality traits, dark triad traits) will likely be a preferred alternative to increasing questionnaire length. Measurement of multiple substantive traits of interest will also likely be a preferred alternative over a separate ERS questionnaire, as no participant time and attention or research funds need to be allocated to measurement of a trait that is not key to the research question at hand. In the case of four substantive dimensions, when aiming to detect ERS at or below – 1.5 or at or above 1.5 four questionnaires of six items seem sufficient. When aiming to detect ERS at or below – 1 or at or above 1, it is advisable to administer four 12-item questionnaires at the least. Overall, the detectability of impactful ERS seems very feasible with reasonable sample sizes and questionnaire lengths in the case of multiple substantive dimensions.

While the current paper provides some guidelines on the use of PPCs for detecting ERS, several limitations and avenues for future research remain. As a first limitation, the present study may not generalize to real-life test conditions fully. While we attempted to maximize generalizability by utilizing item slope parameters estimated from real data, it is likely that researchers attempting to use PPCs in practice may deal with data not originating from any known ERS model, different item parameters, different sample sizes, and a different number of latent traits or correlated substantive latent traits. In addition, the range of conditions, particularly the fixed sample size and the fixed item slopes, was somewhat limited in order to maintain a reasonable computation time potentially further limiting generalizability. Future research would benefit from exploring the impact of these factors on TPR and FPR rates in detecting ERS at both the group and individual levels.

Importantly, researchers are also likely to encounter items with a different number of categories than those examined in this paper. Generally, it is possible for a higher number of categories to enhance the detectability of ERS for two reasons. First, a higher number of item categories may give us more information regarding a participant’s true substantive latent trait value. Second, a higher number of item categories may lead to a lower number of extreme responses occurring naturally due to the substantive trait. Extreme responses may thus become a somewhat stronger indicator of positive ERS, enhancing the detectability of positive ERS values. Of course, this logic may not hold if more responses than the most extreme category are considered extreme responses in the item with additional categories (e.g., for an eight-category scale 1, 2 ,7, and 8 may be considered extreme responses, rather than just 1 and 8). Note that the two reasons listed above assume all else remains equal, which may well not be the case. Increasing the number of categories on a scale may have numerous effects beyond the two listed here, such as introducing noise to the data or potentially increasing the tendency of participants to exhibit response styles. Definitive predictions are thus difficult to make.

As a second limitation, the current research only deals with the detection of ERS using PPCs. Future research may explore the detectability of other response styles, such as the acquiescent response style, the midpoint response style, or perhaps social desirability. Especially data where multiple response styles are present may be of interest to discern potential interaction effects between various response styles when attempting to detect them using PPCs.

As a third limitation, the current research only deals with two PPCs calculated for the GPCM. Since some differences in performance were found for the two proposed PPCs, it is possible that PPCs can be designed that are still more powerful. In addition, the efficacy of these PPCs for different IRT models, such as the graded response model and IRTree models, should be examined.

Overall, the current study showed the potential for two proposed PPCs to detect ERS at both the person and group level without having to fit a response style IRT model or administering a separate ERS questionnaire. The proposed method allows researchers to theoretically justify the use of ERS models without needing to rely on a specific operationalization of ERS, and to detect general model misfit related to extreme responding rather than test for specific operationalizations of ERS being present in the data via model comparisons. Additionally, it frees the researcher to examine other sources of misfit if no ERS-related misfit is found. The need for ERS detection and correction methods was reinforced by the finding that 74 out of 74 countries participating in PISA 2009 showed signs of ERS in the reading enthusiasm questionnaire. While the impact of this ERS was not studied in this paper, the potential of ERS to change substantive conclusions has been demonstrated in other papers (e.g., Moors, 2012; Schoenmakers et al., 2023). Conditions where ERS could be detected in the data were outlined, and practical recommendations for applied researchers wishing to utilize PPCs for ERS detection were made. We encourage researchers to consider the presence of ERS and other response styles in data to avoid bias in their inferences.

## Appendix 1: Detailed convergence strategies

When considering convergence, it is important to consider that in IRT models such as the GPCM, non-convergence may be caused by chains getting stuck in perfectly opposite modes of the posterior. This is due to the fact that flipping the sign of both the item slopes and the person parameters results in identical posterior probabilities and predictions (i.e., ${\alpha }_{i}*\theta +{d}_{ic}=-{\alpha }_{i}*-\theta +{d}_{ic}$). To reduce the incidence of chains getting stuck at opposite points, several remedies can be considered. First, a strict lower bound of zero could be enforced on the item slopes, or a prior assigning zero probability mass to slope values below zero could be used. However, it is very well possible that item slopes of poor items are negative in practice, making such a strong constraint undesirable. As a weaker alternative, one may initialize both chains on the positive side of the posterior (e.g., both chains are initialized with slope parameters $N(5, 1)$). This can be seen as a weak constraint that encourages chains to converge on the positive side of the posterior. If the chains converge to opposite sides of the posterior regardless of the initialization, a final solution may be to multiply the item slope and persons parameters of opposite chains by – 1 and reassess convergence.

In this example, we chose to initialize chains on the positive side of the posterior, and manually reverse the sign of person and slope parameters where necessary. If convergence was not reached after this step, we doubled the number of warmup iterations and reran the model. After, we again manually reverse the sign of person and slope parameters if necessary. If this step still did not lead to convergence, we implemented a lower bound of zero on the slope parameters and reran the model while iteratively increasing the factor by which we multiplied the number of warmup iterations by 1 until the $\widehat{R}$ statistic dropped below 1.01.

In ~84% of the datasets, initializing slopes as $N\sim (5, 1)$ with 1000 burn-in iterations was enough to lead to convergence after manually multiplying opposite chains by – 1 where necessary. In ~14% of the datasets, doubling the number of burn-in iterations was necessary to reach an $\widehat{R}$ below 1.01. In the last ~3% of datasets, a lower bound of zero was implemented and the number of burn-in iterations was iteratively increased to reach convergence. Convergence was reached for all models utilizing this strategy.

## Appendix 2: Comparison of IRTree and GPCM ICC and extreme response probabilities

ICCs (Item characteristic curves) were generated using the GPCM with slopes of 0.5, 1, and 2. GPCM item thresholds of [– 1, 0, 1] were used, with 100,000 participants and ten identical items. The IRTree model without the ERS dimension and the GPCM model were both fit on this data using the mirt R package to obtain item parameters. ICCs of the models appear very close throughout the conditions presented here, which resemble the conditions used in the main simulation study but are somewhat more extreme to ensure models are also equivalent in other conditions.

The models appear near identical for the conditions considered in our simulation study (~1.3 GPCM slope, at the very most – 1.5 threshold shift for the most extreme item, with the mean threshold shift being – 1 at most). Note that this was the case even for the very extreme values of $\theta$, which are very unlikely to occur given $\theta \sim N\left(\text{0,1}\right)$. To further enhance our understanding of the models, we also provide a comparison of the probability of an extreme response (1 or 4) for both models below, using the same conditions as above.
